# Detoxification effects of long-chain versus a mixture of medium- and long-chain triglyceride-based fat emulsion on propafenone poisoning

**DOI:** 10.1590/1414-431X20209491

**Published:** 2020-06-05

**Authors:** Xusheng An, Yong Mei, Hao Sun, Jinsong Zhang

**Affiliations:** 1 Department of Emergency Medicine, The First Affiliated Hospital with Nanjing Medical University, Nanjing, Jiangsu, China; 2 Department of Critical Care Medicine, The Affiliated Huaian No. 1 People’s Hospital of Nanjing Medical University, Huaian, Jiangsu, China

**Keywords:** Long-chain triglyceride-based fat emulsion, Medium- and long-chain triglyceride-based fat emulsion, Propafenone

## Abstract

In the present study, we aimed to compare the detoxifying effects of two fat emulsions containing either long-chain triglyceride or a mixture of medium-chain and long-chain triglycerides in the propafenone-poisoned rat model. Rats were randomly divided into 3 groups according to the fat emulsions used: long-chain triglyceride-based fat emulsion (LL) group; medium-chain and long-chain triglyceride-based fat emulsion (ML) group; normal saline (NS) group. Propafenone was continuously pumped (velocity=70 mg/kg per h) until the mean blood pressure dropped to 50% of basal level. Then, LL/ML fat emulsions or NS was intravenously infused instantly with a loading-dose (1.5 mL/kg) and a maintenance dose (0.25 mL/kg per min) for 1 h. Subsequently, the propafenone was added to plasma (3.5 μg/mL) *in vitro*, mixed with three doses of LL or ML (1, 2, or 4%). Finally, after centrifugation, the concentration of propafenone was measured. Rats treated with LL exhibited accelerated recovery, characterized by higher blood pressure and heart rate. Rats in both the LL and ML groups demonstrated decreased propafenone in plasma (time-points: 15, 25, and 60 min). However, rats that received LL showed lower propafenone in myocardial tissue at the end of detoxification treatment. Rats in the ML group had the lowest value of pH, the minimum content of HCO_3_^-^, and the highest production of lactic acid at the end. In the *in vitro* experiments, propafenone decreased more dramatically in the LL group compared to the ML group. Long-chain triglyceride fat emulsion had a better effect on treating propafenone poisoning in rats.

## Introduction

In recent years, cardiovascular medicine poisoning events have increased because of misuse or suicide. Fat emulsion has been recommended as a treatment in narcotic drug poisonings as well as other drug poisonings based on an international cardiopulmonary resuscitation guideline (1). Fat emulsion was reported to treat cardiovascular medication poisonings such as calcium channel blocker and β-blocker (2), however, the effects of fat emulsion on propafenone poisoning are rarely reported. Propafenone, as a kind of class Ic anti-arrhythmia drug, is widely used. Propafenone poisoning could lead to cardiogenic shock, bradycardia, and even cardiac arrest. A case report showed the potential of fat emulsion in treating propafenone poisoning (3). Therefore, we designed the present animal study to validate the efficacy of fat emulsion in treating propafenone poisoning.

Although long-chain triglyceride-based fat emulsion (LL) draws a lot of attention, medium- and long-chain triglyceride-based fat emulsion (ML) was also reported to successfully treat drug poisoning (4,5). The role of different kinds of fat emulsion in detoxification is controversial, and the effects of fat emulsion in propafenone poisoning are scarcely reported. The present study aimed to investigate detoxification effects of long-chain versus medium- and long-chain triglyceride-based fat emulsion in the propafenone-poisoned rat model.

## Material and Methods

### Animals

Eight- to ten-week-old male Sprague-Dawley rats, purchased from Weitong Lihua Experimental Technology Co., Ltd. (China), were kept in the animal experiment center of Nanjing Medical University with a temperature of 18–25°C, humidity of 30–40%, food and water *ad libitum*, and 12-h circadian cycle. Animals were used for the experiment after being fed for 1 week to adapt to the environment. After the experiments, animals underwent euthanasia via rapid intravenous injection of 1% pentobarbital sodium (150 mg/kg body weight).

### Anesthesia and surveillance of vital signs

All animals were fasted for 12 h before the experiment. Rats were anesthetized with injection of 1% sodium pentobarbital (50 mg/kg). Auxiliary ventilation (tidal volume: 5 mL/kg; respiratory rate: 60 bpm; inhale/exhale ratio: 2:3) was applied by tracheal intubation with a respirator (Model 683, World Precision Instruments Inc, USA).

A median cervical incision was made and the right carotid blood vessels were bluntly separated. Carotid vein intubation was used for propafenone or fat emulsion pumping and carotid artery intubation was used for mean blood pressure and heart rate recording through sensor and transductor connected to Power lab Data Analysis and Processing System (AD Instrument, Australia). The baseline data of blood pressure and heart rate were recorded 5 min after the operation was finished.

### Propafenone poisoning animal model

Thirty rats were randomly divided into 3 groups (n=10/group): long-chain triglyceride-based fat emulsion (LL) group, medium- and long-chain triglyceride-based fat emulsion (ML) group, and normal saline (NS) group. Propafenone was injected (70 mg/kg per h) until the mean blood pressure dropped to half of the baseline value (recorded as 0 min).

### Detoxification

Then, LL, ML, or NS was injected instantly with a loading dose (1.5 mL/kg) and a maintenance dose 0.25 mL/kg per min for 1 h. The heart rate and blood pressure were recorded every 5 min. Meanwhile, 0.5 mL of blood was harvested at 4 time-points (5, 15, 25, and 60 min) for analysis. After blood draw, an equal volume of NS was perfused back into the jugular vein. The whole blood was centrifuged (11,000 *g*) at 4°C for 12 min, and the lower aqueous phase was drawn to measure the concentration of propafenone. Artery blood gas was analyzed at 60 min. Then, the rat was sacrificed and the heart was removed to measure the concentration of propafenone in apical myocardial tissue (400 mg).

### *In vitro* experiment

Propafenone was added to plasma *in vitro* (3.5 μg/mL) and mixed with three doses of LL or ML (1, 2, or 4%). The mixture was incubated for 5 min at a temperature of 37°C and then centrifuged (11,000 *g*) at 4°C for 12 min, and the lower aqueous phase was drawn for analysis. Finally, after centrifugation, the concentration of propafenone was repeatedly measured 3 times.

### Measurement of propafenone concentration

High performance liquid chromatography (Thermo-Fisher Technology Co., Ltd., USA) was used to measure the plasma concentration of propafenone and the concentration of propafenone in myocardial tissue. The parameters of high performance liquid chromatography were: chromatographic column: Phenomenex C18 (4.6 × 100 mm, 2.6 µm); flow rate: 0.6 mL/min; injection volume: 10 µL; column temperature: 25°C. Mobile phase A was methanol, and mobile phase B was an aqueous solution containing 0.1% formic acid. For the plasma concentration of propafenone, 100 µL of water was added and mixed with 50 µL of the lower aqueous phase in the centrifuge tube. Then, 900 µL methanol was added, and the mixture was mixed for 10 min (2500 rpm) by an automatic vortex instrument (MVM-200; Xiangshen Technology, China). Afterward, the mixture was centrifuged (11,000 *g*) at 4°C for 4 min, and the supernatant (0.45 µM) was filtrated by an organic membrane for further analysis.

### Statistical analysis

Statistical analyses were done by GraphPad Prism 5.0 software (USA). One-way or two-way analysis of variance (ANOVA) was applied to campare the difference among the 3 groups. The *t*-test was used for two-group analysis. The data are reported as means±SD. P<0.05 was regarded as a statistically significant difference.

## Results

### Weight, blood pressure, and heart rate at baseline

Rats of the three groups showed no significant difference in baseline body weight, blood pressure, or heart rate (Table 1).

**Figure 1:**
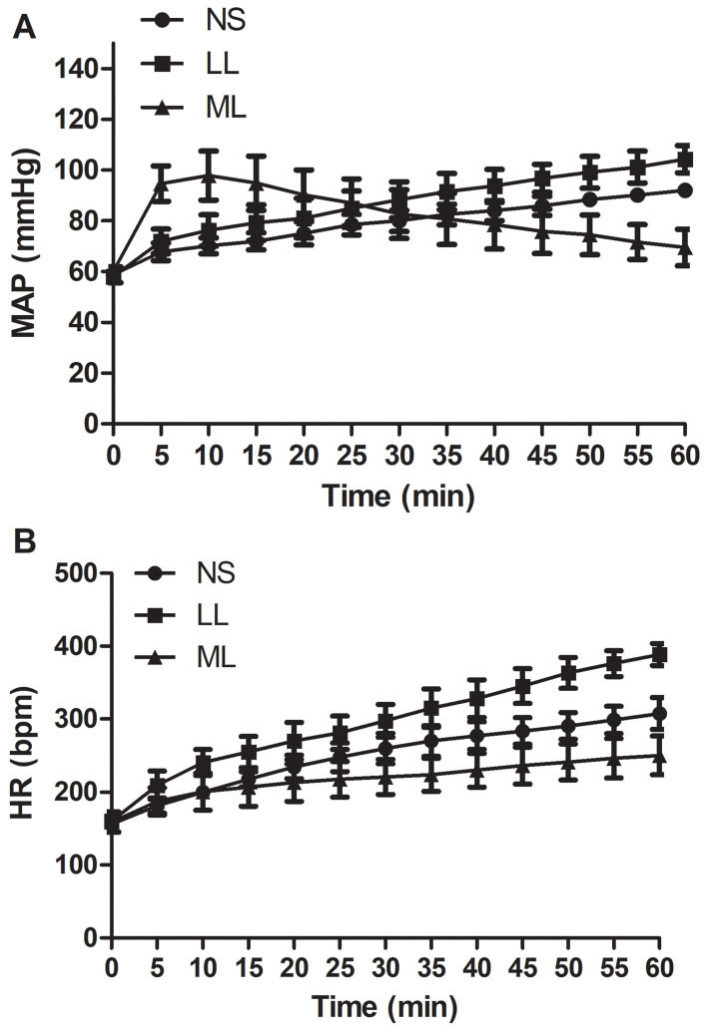
Changes of hemodynamics in the process of detoxification in the propafenone-poisoned rat model. **A**, Mean arterial pressure (MAP). **B**, Heart rate (HR). Data are reported as means±SD. LL: long-chain triglyceride-based fat emulsion; ML: medium- and long-chain triglyceride-based fat emulsion; NS: normal saline.

**Figure 2:**
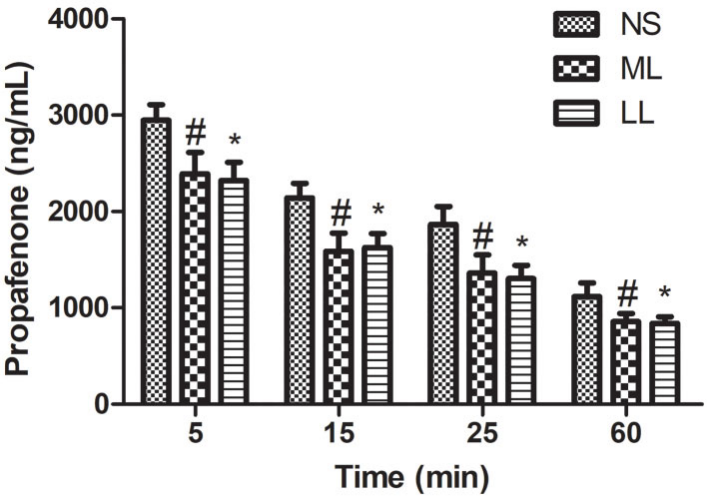
Plasma concentration of propafenone at different time-points. Data are reported as means±SD. *P<0.05, LL compared to NS; ^#^P<0.05, ML compared to NS (ANOVA). LL: long-chain triglyceride-based fat emulsion; ML: medium- and long-chain triglyceride-based fat emulsion; NS: normal saline.

**Figure 3:**
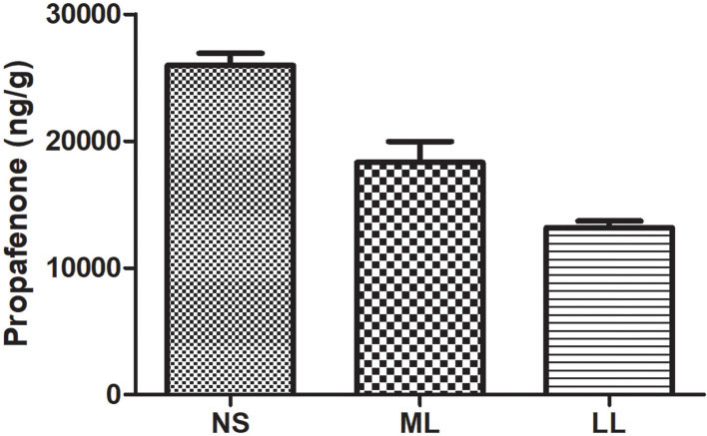
Myocardial tissue concentration of propafenone at different time-points in the propafenone-poisoned rat model. Data are reported as means±SD (ANOVA). LL: long-chain triglyceride-based fat emulsion; ML: medium- and long-chain triglyceride-based fat emulsion; NS: normal saline.

**Figure 4:**
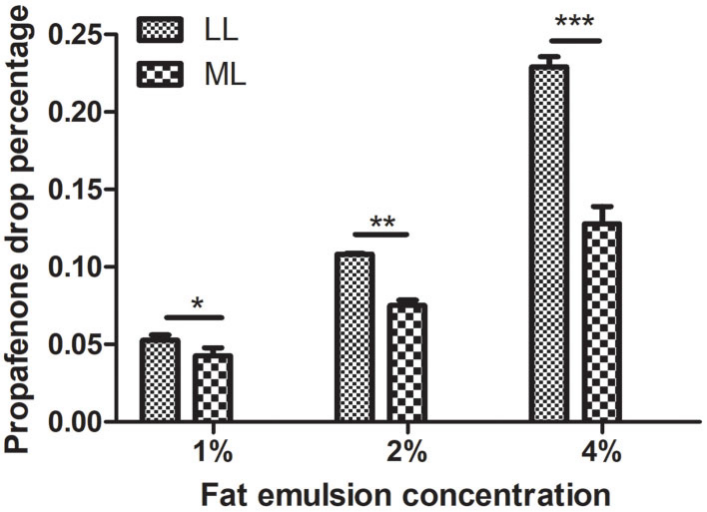
Concentration of propafenone *in vitro*. Data are reported as means±SD. *P<0.05, **P<0.01, ***P<0.001 (*t*-test). LL: long-chain triglyceride-based fat emulsion; ML: medium- and long-chain triglyceride-based fat emulsion.

**Table 1:** Body weight, heart rate, and blood pressure at baseline of the 3 groups in the propafenone-poisoned rat model.

	LL	ML	NS
Weight (g)	331±9	329±8	335±9
HR (bpm)	412±9	409±10	415±9
MAP (mmHg)	109±3	111±2	108±2

Data are reported as means±SD. LL: long-chain triglyceride-based fat emulsion; ML: medium- and long-chain triglyceride-based fat emulsion; NS: normal saline.

**Table 2:** Blood gas analysis among the 3 groups in the propafenone-poisoned rat model.

	Group	Data
pH	LL	7.36±0.03^#^
	ML	7.21±0.04*
	NS	7.31±0.03^+^
PO_2_ (mmHg)	LL	104.9±2.8
	ML	103.6±3.4
	NS	104.4±2.8
Na^+^ (mM)	LL	131.3±3.6
	ML	130.8±3.3
	NS	132.4±4.7
K^+^ (mM)	LL	2.90±0.22
	ML	3.24±0.37*
	NS	3.10±0.28
HCO_3_^-^ (mM)	LL	24.1±1.6^#^
	ML	18.0±2.5*
	NS	21.7±2.1^+^
Lac (mM)	LL	0.31±0.09
	ML	1.79±0.24*
	NS	0.43±0.17^+^

Data are reported as means±SD. *P<0.05, ML compared to LL; ^+^P<0.05, ML compared to NS; ^#^P<0.05 LL compared to NS (ANOVA). Lac: lactic acid; LL: long-chain triglyceride-based fat emulsion; ML: medium- and long-chain triglyceride-based fat emulsion; NS: normal saline.

### Changes of hemodynamics in the process of detoxification

After detoxification treatment, rats injected with LL demonstrated an accelerated recovery, characterized by increased heart rate and blood pressure (Figure 1). However, rats injected with ML exhibited a significant delay of heart rate recovery (Figure 1B). Blood pressure recovery of rats in the ML group was irregular. Blood pressure increased quickly within 5 min, then started dropping 10 min after injection. At the end of the assay, the blood pressure of rats injected with ML was significantly lower than rats in the other two groups (Figure 1A).

### Blood gas analysis

As shown in Table 2, after 60 min of detoxification treatment, the ML group had the lowest value of pH and HCO_3_^-^ and the highest value of lactic acid (P<0.05). The level of potassium was higher in the ML group than the LL group (P<0.05) and NS group (P>0.05). Compared to the NS group, pH value and HCO_3_^-^ concentration were significantly higher in the LL group (P<0.05), while lactic acid concentration tended to decrease in the LL group (P>0.05). PO_2_ and sodium concentration showed no difference among the 3 groups (P>0.05).

### Plasma concentration of propafenone at different time-points

The plasma concentration of propafenone declined in a time-dependent manner. Rats started to display different plasma propafenone concentration 5 min after detoxification treatment (2949±196 ng/mL in NS rats, 2392±171 ng/mL in ML rats, and 2323±163 ng/mL in LL rats). Fifteen min later, plasma propafenone concentration in ML and LL rats declined to 1588±160 and 1625±139 ng/mL respectively, compared to 2140±180 ng/mL in NS rats. Plasma propafenone concentration in ML and LL rats decreased to 1361±150 ng/mL and 1303±116 ng/mL, respectively, 25 min after detoxification treatment. By contrast, plasma propafenone concentration in NS rats was 1866±143 ng/mL. At the end (60 min) of our assay, the plasma propafenone concentration was 857±149 ng/mL in ML rats, 836±113 ng/mL in LL rats, and 1116±14 7ng/mL in NS rats. Our results demonstrated that rats injected with fat emulsion (either LL or ML) had lower propafenone concentration in the plasma at all the time-points tested (Figure 2), compared to rats injected with saline.

### Myocardial tissue concentration of propafenone at different time-points

Rats in the three groups demonstrated significant differences in the concentration of propafenone in myocardial tissue at 60 min (25,957±1830 ng/g in NS rats, 18,310±1960 ng/g in ML rats, and 13,165±1583 ng/g in LL rats, P<0.05). The lowest concentration of propafenone in myocardial tissue was in the LL group and the highest concentration was in the NS group (Figure 3).

### Concentration of propafenone *in vitro*

The degree of propafenone decrease was higher in the LL group, compared with the ML group (2% LL *vs* 2% ML: 0.1082±0.0006 *vs* 0.075±0.005; 4% LL *vs* 4% ML: 0.2291±0.0093 *vs* 0.1277±0.0161, P<0.01), in the *in vitro* experiment (Figure 4).

## Discussion

In the present study, a propafenone-poisoned rat model was set up to compare detoxification effects between LL and ML. From this study, LL performed well to treat propafenone poisoning by improving heart rate and blood pressure. Compared to the NS group, the plasma concentration of propafenone at 5, 15, 25, and 60 min was lower in LL group. The concentration of propafenone in myocardial tissue was significantly lowered at 60 min in the LL group. In spite of decreasing the concentration of propafenone in plasma and myocardial tissue, ML failed to improve the hemodynamic parameters.

Russell and Westfall reported that fat emulsion could shorten anesthesia duration under thiopental sodium, indicating fat emulsion might have a detoxification effect on lipophilic drug (6). Inspired by the above-mentioned study, Weinberg et al. (7) verified the detoxification effect of fat emulsion on bupivacaine poisoning. The half lethal dose of bupivacaine was elevated to 18.5 from 12.5 mg/kg under the treatment of fat emulsion. Another study found a lethal dose of bupivacaine could lead to death in a canine model, however, the experimental dog survived from a lethal dose of bupivacaine after fat emulsion treatment (8). Fat emulsion could rapidly decrease the concentration of bupivacaine in myocardial tissue, and the function of cardiomyocytes was improved in the meantime, based on a bupivacaine poisoning-induced cardiac arrest rat model (9 [Bibr B10]). Consequently, fat emulsion could be used as a detoxification reagent to treat lipophilic drug poisoning underlying the lipid pool theory. Fat emulsion in plasma could form a lipid pool, which could adsorb a lipophilic drug and eliminate it from tissue and cell. As a result, the concentration of the drug could decrease, and its toxicity mitigated.

The effects found in this study were related to the combination between fat emulsion and propafenone, resulting in a concentration gradient of propafenone between myocardial tissue and blood, prompting the translocation of propafenone from tissue to blood, and alleviating cardiac mechanic and electronic dysfunction. Therefore, the theory of lipid pool was further strengthened.

Fat emulsion was mainly applied to treat lipophilic drug poisoning, and lipid-water partition coefficient could be used to predict the efficacy of fat emulsion on cardiac toxicity (11). Mazoit et al. (12) found that bupivacaine and ropivacaine had different lipid-water partition coefficients, and the capacity to combine with fat emulsion was better in bupivacaine than ropivacaine. They also found that the capacity of the local anesthetic drug bupivacaine combined with LL was 2.5 times higher than that in ML. However, Ruan et al. (13) hold different viewpoints, and they suggested ML performed better than LL in combination with a local anesthetic drug in the *in vitro* study. Our data showed that LL could combine better with propafenone than ML *in vitro*. In the *in vivo* study, fat emulsion (both LL and ML) could significantly decrease the concentration of propafenone at each time-point compared to NS, and LL could further eliminate even more propafenone than ML did. The concentration of propafenone in myocardial tissue was lower in the LL group than in the ML group, indicating LL performed better than ML in combination with propafenone.

The detoxification mechanism of fat emulsion strengthened fatty acid metabolism and improved cardiac function. After metabolizing, fat emulsion is transformed into fatty acid. As the main source of myocardial energetic embolism, fatty acid could supply cardiomyocytes and improve cardiac function (14,15). ML with a small molecular weight could be translocated into cardiomyocytes and mitochondria through simplified diffusion, independent from carnitine transferase (16). Besides, ML had a better effect in improving cardiac function (17). Thus, our animals should have benefited more from ML treatment, theoretically.

In the present study, compared to LL, less propafenone was combined to ML; however, the propafenone concentration in the ML group was significantly lower than that in the NS group. Regardless of the capacity of propafenone clearance, the heart rate was slower in the ML group than that in the LL group or NS group. Besides, in the early phase of detoxification (5-10 min), blood pressure was elevated promptly for a while. After reaching the peak, although the blood pressure slowly declined in the ML group, its value was still higher than the one in the LL group or NS group within 25 min. Nevertheless, the gradually decreased blood pressure in the ML group was lower than that in the LL group or NS group after 30 min. Even acidosis, increased lactic acid, and hyperkalemia took place in the ML group after 60 min.

The reasons why ML failed to treat propafenone poisoning are listed as follows. Myocardial ischemia and hypoxia lead to strengthened glycolysis and subsequent cellular acidosis. At the same time, free fatty acid is mobilized to supply the need of energy. Compared to glucose oxidative decomposition, more oxygen is needed for fatty acid oxidation, and this could accentuate myocardial hypoxia (18). Acetyl CoA, the β-oxidation of fatty acid, could inhibit the activity of pyruvate dehydrogenase and the decomposition of pyruvate, leading to increased glycolysis and cellular acidosis (19). Injection of ML offered lots of medium-chain fatty acid, increasing cardiac energy supply, strengthening myocardial contractility, and elevating blood pressure in a short time. However, impaired glucose oxidation, accentuated cellular acidosis, and damaged cardiomyocyte function resulted in a gradual decrease of blood pressure. Since the entrance of long-chain fatty acid into cardiomyocytes is limited by carnitine acyltransferase, injection of long-chain fatty acid had little effect on myocardial energetic metabolism and blood pressure.

In the early phase, the injection of ML leading to rapid elevation of blood pressure was consistent with a previous study (19), and this might be related to increased intracellular calcium and strengthened left ventricular contractility. Calcium overload originating from myocardial ischemia could interfere in mitochondrial oxidative phosphorylation. ML could promote calcium influx and aggravate cellular calcium overload, leading to disorder of myocardial energetic metabolism and cardiac dysfunction. Fat emulsion was used to treat cardiovascular medication poisoning such as calcium channel blockers (20–22), possibly owing to increased intracellular calcium, increased cardiac contractility, and alleviated calcium channel blocker toxicity.

Limitations of the present study included: 1) the concentration of propafenone in fat emulsion should have been measured to directly reflect the elimination of propafenone; and 2) after drawing blood repeatedly, normal saline supplementation could have led to dilution of propafenone, and this might influence the measurement of the propafenone concentration.

We conclude that LL had a better effect in treating propafenone poisoning; however, ML did not show detoxification effects on propafenone poisoned rats. Detoxification effects of LL in propafenone poisoning might be due to the lipid pool theory.
